# Financing drug development via adaptive platform trials

**DOI:** 10.1371/journal.pone.0325826

**Published:** 2025-07-02

**Authors:** Joonhyuk Cho, Eugene Sorets, Shomesh Chaudhuri, Annette De Mattos, Kristin Drake, Merit E. Cudkowicz, Ricardo Ortiz, Meredith Hasenoehrl, Marianne Chase, Brittney Harkey, Sabrina Paganoni, John Frishkopf, Andrew W. Lo

**Affiliations:** 1 MIT Laboratory for Financial Engineering, Cambridge, Massachusetts, United States of America; 2 Questrom School of Business, Boston University, Boston, Massachusetts, United States of America; 3 QLS Advisors, Cambridge, Massachusetts, United States of America; 4 Sean M. Healey & AMG Center for ALS at Massachusetts General Hospital, Boston, Massachusetts, United States of America,; 5 MIT Sloan School of Management, Cambridge, Massachusetts, United States of America,; 6 MIT Computer Science and Artificial Intelligence Laboratory, Cambridge, Massachusetts, United States of America,; 7 Santa Fe Institute, Santa Fe, New Mexico, United States of America; Kings College Hospital, UNITED KINGDOM OF GREAT BRITAIN AND NORTHERN IRELAND

## Abstract

We propose a new approach to funding disease-specific drug development via a variation of the adaptive platform trial. This trial is designed to test a portfolio of drug candidates in parallel, with the cost of the trial partially covered by investors who receive payments from a royalty fund of the candidates in exchange for investment. Under realistic assumptions for cost, revenue, probability of success, drug sales, and royalty rates, investors may expect a return of 28%, but with a 22% probability of total loss. Such return distributions may be attractive to hedge funds, family offices, and philanthropic investors seeking both social impact and financial return. Return distributions palatable to mainstream investors may be achieved by funding multiple platform trials simultaneously and securitizing the aggregate cash flows.

## Introduction

The randomized clinical trial (RCT) process for developing a novel therapeutic agent is notoriously expensive and lengthy, and the outcomes are often highly uncertain. Several methods have been proposed to lower the expense and duration of clinical trials using innovative design, and one of the most effective is the adaptive platform trial (APT) [1–3]. This approach involves testing multiple investigational products simultaneously for a single disease and interpreting the results on a real-time basis using Bayesian statistics to determine efficacy or futility. Although APTs are more complex to manage than traditional fixed-sample two-arm RCTs, their design—with a master protocol, parallel testing, and a shared placebo group—has attracted interest. A simulation study demonstrated that an APT design, compared to a series of 10 sequential two-arm trials, resulted in approximately a 76% reduction in decision time and median cost savings of about 37% [4]. Despite these benefits, clinical trials are still costly—requiring tens to hundreds of millions of dollars per trial—and funding gaps can delay the launch of a trial by years.

In this paper, we propose a new approach to address the funding gap through the use of an investment fund that pays for part of the cost of an APT in exchange for future royalties on the drugs included in the trial. Using data from the APT administered by the Healey & AMG Center for ALS at Massachusetts General Hospital, we find that this fund of adaptive royalties (FAR) would generate an internal rate of return (IRR) of 28% on average, but with a probability of total loss of 22%.

Such a risky return distribution might not be attractive to mainstream investors, but it could be of considerable interest to more risk-tolerant and mission-driven investors, including hedge funds, family offices, sovereign wealth funds, and patient advocates engaged in venture philanthropy.

## Literature review

The advantages of platform clinical trials to patients and researchers have been discussed in several studies [1–6]. Several authors in the earlier literature have proposed enlarging the pool of potential investors for drug development by creating financial instruments that would transfer its inherent risk, and thereby reduce it. In particular, the idea of a drug development megafund—a fund that would diversify the risk by investing in a large number of biomedical assets—was initially proposed [7], later extended [8], and further specialized for orphan disease drug development [9,10] or further innovations [6]. Another method to transfer the risk of drug development was put forth, where the authors created the concept of an “FDA Hedge” [11], an instrument that would allow drug companies to exchange some of their (potential) future revenues for cash in early development. A similar type of risk transfer has, in fact, been implemented by Royalty Pharma [12].

The megafund approach can be further enhanced by securitization, i.e., by separating the cash flows generated by successful programs in the megafund into “tranches” that can be packaged as individual bonds at a given credit rating [8]. The senior tranches can be made to have very favorable risk characteristics to appeal to mainstream investors, while the junior tranches contain greater risk and greater return, appealing to hedge funds and other specialist investors. The idea of securitizing pools of individual cash flows is, of course, far from new; it has been used for decades for mortgages, student loans, and other sources of cash flow, but has yet not been used for financing drug development.

## Modeling of clinical trials

We model a standard APT running five batches of four independent trial regimens, where a batch refers to a cohort of four investigational arms launched under a single master protocol with one-month intervals between regimen starts. The design mirrors the HEALEY ALS Platform Trial schedule [5,13,14]. Each regimen in our model represents a phase 2/3 trial, with all regimens having passed phase 1 and focused on ALS treatment.

Key parameters in our model include trial cost, duration, probability of success, market size for approved drugs, and regimen enrollment scheduling. Below, we describe these parameters and their calibration in more detail. Summarized key input parameters are listed in Table 1.

**Table 1:** Key input parameters for Monte Carlo simulation of the royalty fund.

Model Parameter	Value	Explanation
**Cost Sharing**	50%	Trial cost sharing percentage between drug developers and the Adaptive Platform Trial Fund
**Percent Royalty**	7%	Royalty provided to the Adaptive Platform Trial Fund
**Regimen Trial Duration**	37 months	Duration of phase 2/3 trials
**Regulatory Review/Approval Duration**	1 year	For successful drugs, time required for NDA/BLA approval before launch
**Interval within Batch**	1 month	Refer to Fig 1
**Interval between Batches**	15 months	Refer to Fig 1
**Number of Regimens in Batch**	4	Refer to Fig 1
**Probability of Success (PoS)**	15.0%	Combined PoS of Phase 2, 3, and NDA/BLA in ALS (Citeline)
**Inflation Rate**	2.6%	According to BRDPI projection [15]
**Fixed Initial Platform Cost**	$2.26 MM	One-time cost for building the platform
**Shared Costs per Batch**	$6.93 MM	Cost of trial operations of each batch; shared across 4 regimens
**Cost per Regimen**	$19.49 MM	Cost of trial operations of each regimen, not including shared costs
**Platform-Based Correlation**	20%	Refer to S1 Fig
**Methodology-Based Correlation**	80%	Refer to S1 Fig
**Disease-Based Correlation**	10%	Correlation between platform trials and non-platform trials; used to account for competitor effects
**Price of the Drug**	$168K	According to the pricing of Relyvrio [16]
**Discount Rate**	13.69%	Cost of capital for biotechnology company of 9.05% [17] with risk-free rate of 4.64% [18]
**ALS Prevalence (US)**	24,309	With growth rate of 1.2% [19]
**ALS Prevalence (EU)**	31,100	With growth rate of 0.7% [19]
**ALS Prevalence (RoW)**	33,000	With growth rate of 1.4% [19]
**Peak Market Share**	55%	Peak market share when regimen is approved [16]

This table summarizes the principal model inputs used to simulate the financial performance of the 50% investment, 7% royalty adaptive platform trial fund. Parameters include cost-sharing and royalty rates, trial scheduling (batch size and timing), phase-transition probabilities, inflation and discount rates, regimen-specific costs, platform and methodology correlations, and drug pricing assumptions. Values are drawn from the HEALEY ALS Platform Trial data and published sources (see text and Supporting Information for details).

**Fig 1:**
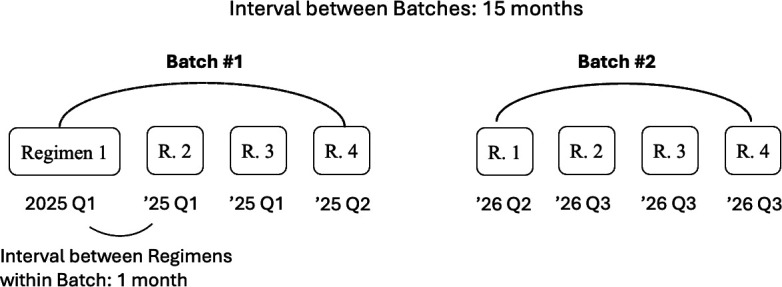
Scheduling of regimen enrollment for platform trials. The figure shows the planned schedule for regimens in Batch #1 and Batch #2, each consisting of four regimens. The diagram highlights the one-month interval between regimens within a batch and the 15-month interval between the start of each batch. Our simulation includes a total of five batches.

### Clinical trial costs, duration, and probability of success

To estimate trial costs, we use data from the 2019 HEALEY ALS Platform Trial, adjusted for inflation to 2025 dollars. This assumes an orally-administered drug tested in 160 participants across 54 clinical sites, including a 6-month follow-up during the RCT period and an overlapping Active Treatment Extension (ATE) [20–24].

Platform trial costs are divided into platform build, trial design, and trial operation. Platform build costs are one-time expenses for infrastructure, trial design costs cover early-stage development, and trial operation costs include running the trial, with shared and fixed components per regimen [13,14]. Based on data from the HEALEY ALS Platform Trial, our model estimates platform build costs at $2.26MM (adjusted to 2025 dollars), with the addition of one regimen costing $21.2MM over 37 months. Post-2025, a 2.6% annual inflation rate is applied based on BRDPI projections [15].

Trial duration is assumed to be 37 months for phase 2/3 trials, which is shorter than the typical 6.7 years [25] for sequential phase 2 and phase 3 trials, benefiting both patients and investors. It is important to note that such efficiencies can be achieved through combined phase 2/3 designs, not just adaptive platform trials. Reflecting the HEALEY ALS Platform Trial data, our default regimen sample size is 160 participants; in the first batch, actual enrollments were 164, 167, 161, and 163 for Regimens A–D. Each RCT participant was followed for 24 weeks, with six to seven in-person visits plus three brief phone check-ins. Subsequent ATEs included six in-person visits and five brief phone check-ins over a 52-week period assuming a 10% drop-out rate from the RCT period (*n* = 144 ATE participants) [21–24]. If successful, an additional year is required for NDA or BLA approval before launch [26].

The probability of success (PoS) for phase 2/3 trials is based on Citeline’s Pharmaprojects and TrialTrove databases for historical ALS trials, with phase 2 at 54.5% (12 successes among 22 trials), phase 3 at 38.5% (5 successes among 13 trials), and NDA/BLA at 71.4% (5 successes among 7 trials), resulting in a combined PoS of 15.0%. This PoS is used in the main analysis, with a sensitivity range from 5% to 25%. 

Detailed cost breakdowns and inflation adjustments are provided in the S1 Appendix.

### Market size

Epidemiological projections estimate the 2025 ALS prevalence at 24,309 in the US, 31,100 in the EU, and 33,000 in rest of the world, with annual growth rates of 1.2%, 0.7%, and 1.4%, respectively [19]. Market data for Exservan™ show that 70% of ALS patients are prescribed riluzole [27], which we use to estimate the treatable market size.

For modeling new drug market share, we use the forecast for Relyvrio™ as a baseline, despite its discontinuation. We set a peak market share at 55% [16], with a three-year linear ramp-up from 0%, and assume loss of exclusivity 12 years post-launch, resulting in a 50% annual market share decline until stabilizing at 10% of the peak. Although our current model does not incorporate the potential effects of IRA price negotiations after 9 years for small molecules, we recognize this could further impact market share trajectories and plan to explore this factor in future analysis.

The drug price is estimated from Amylyx’s Relyvrio™ data, set at $168,000 annually for 2025 (inflated from $158,000 in 2023 using a 6.1% BRDPI adjustment). A 2.6% annual price increase is applied per BRDPI projections, with a compliance rate of 85%.

### Platform trial: regimen enrollment scheduling

The model platform trial proceeds in batches, with each batch consisting of four independent regimens. This structure is taken from the HEALEY ALS Platform Trial, which enrolled 4 regimens at a time starting in 2020 [5].

Accordingly, in our model, a new batch is added to the platform trial every 15 months as described in Fig 1. We assume that the platform trial will continue for five batches, encompassing a total of 20 different regimens. This batch scheduling is based on the feasibility and capacity of the HEALEY ALS Platform Trial, which has demonstrated the capacity to process up to 12 regimens concurrently. The first batch in our model is assumed to enroll in January 2025.

## Financial structure of the fund

In this section, we describe the structure and operational strategy of our model’s royalty fund, which is specialized to finance a portfolio of clinical trials belonging to a single platform.

We calculate the potential net present value (NPV), IRR, and number and timing of drug approvals via Monte Carlo simulation [6] with Excel. For the discount rate, we use 13.69%, based on the real cost of capital for biotechnology companies of 9.05% [17] and the risk-free rate of 4.64% [18]. Additionally, we account for pairwise correlations between trials, and consider the effects when multiple drugs (including those by competitors) are approved and available in the market.

The royalty fund operates by subsidizing a certain percentage of platform trial costs to reduce the financial burden on drug developers. In our baseline model, this subsidy is set at 50%. Consequently, during the early stages, the fund experiences negative cash flow until the first drug in the portfolio is approved. Once a drug receives approval, the fund earns a percentage of the drug sales through royalty ownership, compensating for the initial investment in trial costs. In our baseline model, this royalty percentage is set at 7%.

For simplicity and greater transparency, we do not incorporate debt financing or other financial techniques in this analysis. Instead, we focus solely on evaluating the financial feasibility and profitability of the royalty fund by examining the distribution of NPV and IRR, based on 10,000 Monte Carlo simulations. The number of simulations, 10,000, is selected to ensure the standard error of the mean NPV remains below 1%, providing a high degree of accuracy in the Monte Carlo analysis.

### Competitor effects on market share

Currently, ALS treatment options are limited, with riluzole and edavarone being the only FDA-approved drugs [28]. Therefore, we consider that the first approved ALS drug in our portfolio will be able to replace or co-exist with them without facing significant competition. Given the prevalence of riluzole and edavarone prescriptions, the initial market entry is assumed to capture a substantial share of the treatable population.

However, for subsequent drugs approved in the portfolio, we must account for competitive effects. When two or more drugs in our model are approved, we assume that the more recently approved drug will become the market leader, while the previously approved drugs’ market share will decay. This involves assessing market dynamics such as the introduction of new therapies, changes in treatment guidelines, and shifts in patient and physician preferences. By incorporating these factors, we aim to provide a realistic estimate of market share that reflects competitive pressures in the biopharma industry.

To model the market share decay after a competitor enters the market, we treat the emergence of a competitor as analogous to the loss of exclusivity due to patent expiration. The impact of a competitor on market share begins the year following the competitor’s emergence. Consequently, in our model, the loss of exclusivity for each program is defined by the following:


$${LoE}_{i}\;=\;\min({App}_{i}\;+\;12,{App}_{j}\;+\;1)$$ (1)

where *LoE*_*i*_ is the year of loss of exclusivity for the *i*th regimen, *App*_*i*_ is the year of approval for the *i*th regimen, and *App*_*j*_ is the year of approval for the *j*th regimen, the regimen approved immediately after the *i*th regimen.

To ensure that total market share does not exceed realistic limits, we apply a normalization process. When initially setting the peak market share for each regimen and considering multiple potential approvals, the total market share might exceed 100%. To address this, we normalize the market shares so that their total does not surpass a predefined overall market share. The baseline value for this overall market share is set at 100%, but it can be adjusted to higher values, up to 300%, to account for scenarios where patients may be prescribed multiple drugs simultaneously. For an existing competitor’s treatment, e.g., Relyvrio™, we assume it will retain at least a 20% market share due to its current high prevalence, even after multiple new regimens are introduced. Therefore, the normalization factor and the normalized market share will be determined by the following:


$$Norm=\;\frac{min(Total\;Share-20\;\%,\;\;\;\sum_{i\in \left\{Healey\cup Compete\right\}}Market\;Share_{i})}{\sum_{i\in \left\{Healey\cup Compete\right\}}Market\;Share_{i}}$$ (2)

$${Normalized\;Market\;Share}_{i}\;=\;{Market\;Share}_{i}\;\times \;Norm$$ (3)

### Correlation between regimens

Some regimens are inherently correlated due to similarities in their targets or the technologies they use. To be conservative, we assume a systematic pairwise correlation exists even if their specific technologies differ (assuming no correlation would yield more attractive risk-adjusted returns). We vary these correlation values in our sensitivity analysis to examine how other correlation scenarios affect the performance of the fund.

Similarly, we assume that all regimens in our platform trial inherently have a platform-based systemic correlation. Between our platform regimens and competitor regimens, we assume there exists a disease-based correlation, which is lower than the platform-based systemic correlation, as they only share the same target disease without the same platform technology. Finally, some regimens within our trial exhibit a methodology-based correlation due to similarities in their drug development methodologies, which we will assume to be high.

The percentage of strong correlations among platform regimens is determined by the results of the HEALEY ALS Platform Trial. From the trial, we can observe that 2 regimen pairs (e.g., regimens 1 and 2, regimens 3 and 4) among the 7 regimens are highly correlated, or, in a pairwise view, that 2 pairs out of 21 pairs are highly correlated. We align our model—which includes 20 regimens with 190 pairwise correlations within the platform—with the HEALEY data by setting 20 out of 190 pairwise correlations to be highly correlated. For the baseline model, we assume a disease-based correlation of 10%, platform-based systemic correlation of 20%, and methodology-based correlation of 80%. The correlation map for platform regimens is displayed in S1 Fig.

## Results

**Fig 2:**
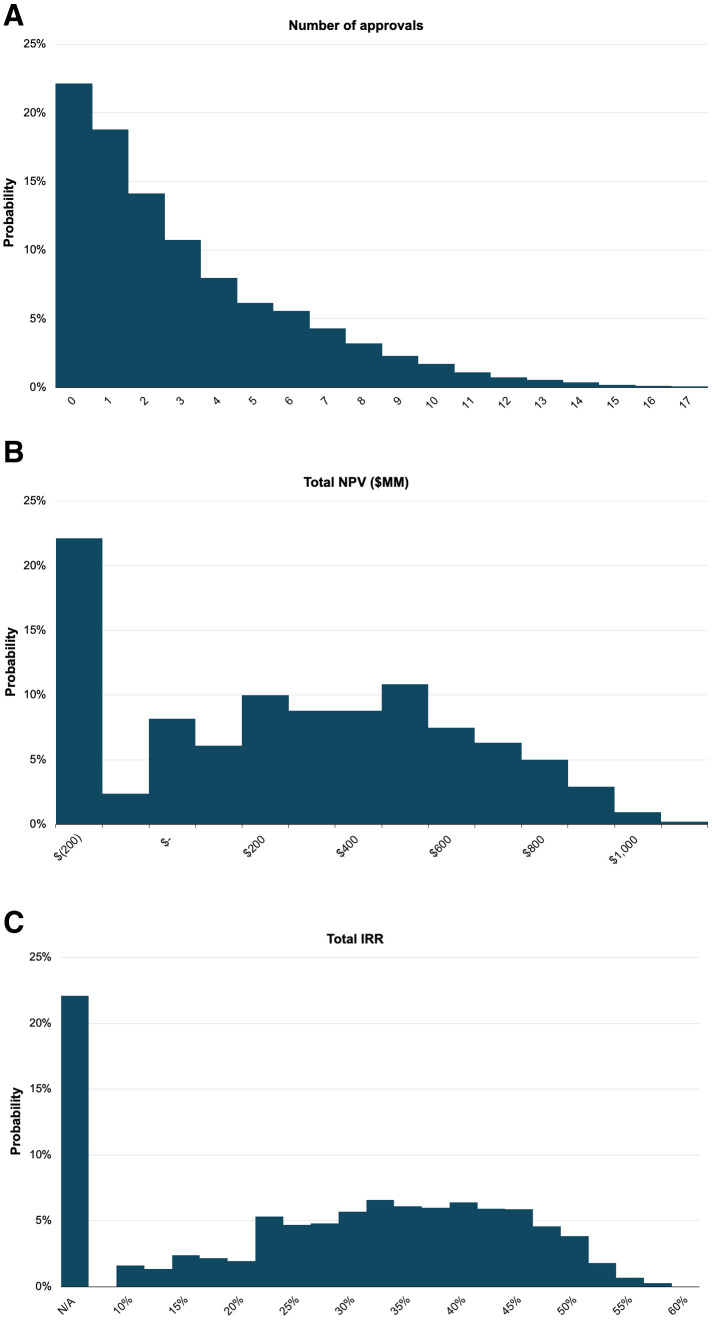
Statistical distributions of the fund’s key performance indicators from 10,000 runs of Monte Carlo simulations. The figure illustrates probability distributions for three key performance indicators: (a) the number of drug approvals, (b) the net present value (NPV) in millions of dollars, and (c) the internal rate of return (IRR). Each distribution represents the results from 10,000 simulation runs. (c) includes cases with “N/A” IRR values, corresponding to scenarios with no approvals.

**Fig 3:**
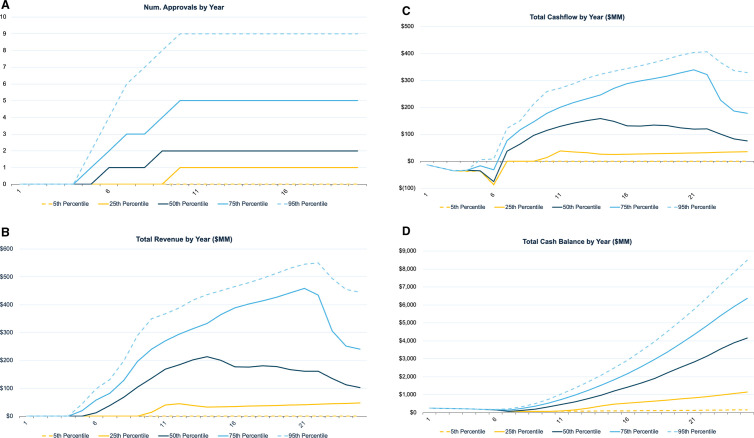
Cumulative number of drug approvals and projected financial metrics over time from Monte Carlo simulations. Panel (a) shows the cumulative number of drug approvals over time with percentile lines (5th, 25th, 50th, 75th, and 95th). Panels (b), (c), and (d) display annual projections for revenue, cash flow, and cash balance, respectively. Each panel demonstrates the variability in outcomes under different approval scenarios, highlighting the distribution of potential financial metrics.

We conducted a Monte Carlo simulation with 10,000 replications to evaluate key performance indicators, including the IRR, NPV, cashflow, cash balance, revenue, and number of drug approvals over time. Table 2 and Figs 2–3 contain the results of the simulation, including investment performance metrics, probability distributions, and percentiles for these indicators, highlighting the range of possible outcomes and their associated likelihoods. In this simulation, the royalty fund starts in January 2025 with $250MM in assets with a time horizon of 25 years, through 2049.

**Table 2:** Statistical summary of key performance indicators for the royalty fund.

	Mean	Stdev	Min	5% pct.	25% pct.	Median	75% pct.	95% pct.	Max
**Num. Approvals**	**3.1**	3.1	0.0	0.0	1.0	**2.0**	5.0	9.0	18.0
**Net Present Value ($MM)**	**$300.70**	$341.22	−$151.33	−$151.33	−$16.17	**$302.03**	$575.56	$857.73	$1,131.45
**Internal Rate of Return**	**N/A**	N/A	N/A	N/A	12.3%	**28.4%**	38.3%	47.4%	56.2%

The table displays the mean, standard deviation, minimum, 5^th^ percentile, 25^th^ percentile, 50^th^ percentile, 75^th^ percentile, 95^th^ percentile, and maximum values for the number of approvals, net present value (NPV), and internal rate of return (IRR). The 50^th^ percentile values are highlighted to show the mean and median expected outcomes for each metric. N/A values in IRR are due to no approval, and the mean calculation of IRR is excluded from our analysis.

### Statistical distributions and key performance indicators

Table 2 and Fig 2a show that the mean number of approvals is 3.1, with a standard deviation of 3.1. The distribution of approvals is highly skewed, with a 22.1% chance of obtaining zero approvals. However, there is also a substantial probability of achieving multiple approvals, with the 95th percentile reaching 9 approvals and a maximum of 18 approvals. The number of approvals at the 50th percentile (the median) is 2. This skew is influenced by the systemic and methodological correlations between regimens.

Fig 2b shows the probability distribution of NPV, which has a large mode in the left tail—the 5th percentile is −$151.33MM, consistent with the worst 22.1% of scenarios of no approval. The 95th percentile reaches $857.73MM, highlighting the potential for outsized returns in the best-case scenarios. The mean NPV is $300.70MM and the median (50th percentile) is $302.03MM, implying a profitable outcome for average and median fund performance.

Fig 2c contains the probability distribution of the IRR for the royalty fund. It is important to note that in 22.1% of the simulations there is no return due to the absence of approvals, and hence, the IRR is undefined in these cases. Therefore, IRR is excluded from the mean and standard deviation calculations shown in Table 2. The 25th percentile is at 12.3%, the median is at 28.4%, and the 75th percentile is at 38.3%, with the maximum at 56.2%.

The median IRR is very high, but the probability of total loss is also high, likely making this return distribution attractive only to sophisticated investors. Using financial engineering methods such as a megafund structure and securitization as described in previous works [7–10] could make some of the cash flows attractive to mainstream investors as well. In Sensitivity Analysis, we find that the results of our simulation are generally robust over a reasonable range of parameter values such as trial success rates, drug prices, and market competition.

### Financial analysis over time

Fig 3a illustrates the projected cumulative number of drug approvals over time, which plays a crucial role in determining the fund’s overall performance. A higher number of approvals directly correlates with increased potential revenue generation, significantly impacting the fund’s financial outlook. Revenue projections (Fig 3b) show that revenue in the most optimistic scenario for the royalty fund can start coming in as early as 2029, while in the least optimistic scenario, it starts in 2034. However, in the worst-case scenario, which occurs with 22.1% frequency in our simulations, no revenue is generated due to the absence of drug approvals. In our model, the first batch of platform trials in the portfolio is set to begin its phase 2/3 trial in January 2025.

Predicted cash flows (Fig 3c) are consistent with these revenue projections—there is no revenue and only cash outflows for trial costs during the first five years, resulting in negative cash flow until 2029 across all scenarios. However, once a drug is approved, the revenue generated from the approved drug surpasses trial costs, leading to positive cash flow beginning between 2029 and 2034 for the 25th and higher percentiles of outcomes.

Similarly, the cash balance in Fig 3d, which starts with an initial investment of $250MM, shows a significant increase after 2030 if any drugs are approved. Even in the scenario where no drugs are approved, this initial $250MM starting balance is sufficient to cover the fund’s 50% investment on the total $426.46MM cost (2025 dollars) of the 20 trials (see S1 Appendix for detailed cost breakdown). By capping the fund’s support at 50%, we ensure substantial financial commitment from drug developers, thereby aligning their incentives with accelerated development timelines and rigorous cost control.

### Sensitivity analysis

To assess the robustness of our findings, we have conducted sensitivity analysis on the key model parameters. Table 3 quantifies how varying the PoS from 5% to 25% influences the royalty fund’s performance. As PoS rises, the fund’s default ratio falls sharply (from 58.1% at 5% PoS to 8.0% at 25% PoS), median NPV increases substantially (from −$151.3MM to $394.1MM), median IRR improves (from undefined in the no-approval scenario to 31.9%), and the median number of drug approvals grows linearly (from 0 to 4). It is noteworthy, however, that NPV growth is not proportional to the rise in approvals—because we cap total market share, additional approvals do not directly translate into a linear increase in net sales or NPV. Moreover, as overall PoS increases, the likelihood of competitor approvals also rises, which can further constrain market share and dampen NPV growth. Additional sensitivity analyses—exploring platform-based correlation, peak market share, discount rate, and drug price—are provided in Tables S2–S5 in S1 Appendix Supporting Information.

**Table 3:** Sensitivity of key performance metrics to probability of success (PoS).

PoS	Default Ratio	Median NPV ($MM)	Mean (Std) NPV ($MM)	Median IRR	Median Approval	Mean (Std) Approval
**5%**	58.1%	−$151.33	$81.62 (319.09)	**N/A**	0	0.9 (1.6)
**10%**	34.4%	$206.06	$219.23 (345.78)	**23.4%**	1	2.0 (2.5)
**15%**	**22.1%**	**$302.03**	**$300.70** **(341.22)**	**28.4%**	**2**	**3.1 (3.1)**
**20%**	14.9%	$374.12	$353.89 (328.41)	**31.4%**	3	4.1 (3.6)
**25%**	8.0%	$394.08	$377.44 (293.30)	**31.9%**	4	4.7 (3.8)

This table shows how varying the probability of success (PoS) from 5% to 25% affects the royalty fund’s default ratio, median and mean net present value (NPV; with standard deviation in parentheses), median internal rate of return (IRR), and median and mean number of drug approvals (with standard deviation). The 15% PoS row (highlighted) represents our baseline scenario.

## Discussion

### Limitations and considerations for broader application

Our current drug pricing assumption predominantly reflect US market dynamics specifically based on the pricing data of Relyvrio the most recent ALS treatment pricing available. Due to the absence of comparable pricing information from other regions, we uniformly applied this US pricing across all markets in our model. However, to better reflect regional market differences, we explicitly incorporated a three-year delay for the European market launch compared to the US, considering standard regulatory and market access timelines. This delay accounts for typical EU-specific processes such as CHMP evaluation (standard duration approximately one year), national health technology assessments, reimbursement negotiations, and other administrative procedures.

In our model, drug pricing was kept uniform across different regions. However, we conducted a sensitivity analysis to evaluate the impact of varying drug prices. Our sensitivity analysis holds patient demand fixed across price scenarios. We did not model price–demand elasticity because there are currently very limited treatment options for patients with ALS and demand for any approved therapy is effectively inelastic. Nonetheless, in disease areas with more therapeutic alternatives or broader patient populations, demand may respond to price changes. Future work could extend our framework by coupling price–demand curves with revenue projections, thereby examining how lower-priced but higher-volume drugs would alter fund performance.

Furthermore, it is noteworthy that our simulations employ a lower PoS for phase 3 trials compared to phase 2, which appears counter-intuitive given the typical trend of increasing success rates in later clinical stages [25]. However, this unusual relationship arises specifically from our reliance on ALS-specific historical data, reflecting the distinct clinical complexities, endpoint variability, and patient heterogeneity inherent in ALS trials [29]. For applications of our model to other diseases, employing disease-specific historical PoS values would provide more realistic and tailored financial estimates.

Even though this study focuses on ALS using parameters derived from the HEALEY ALS Platform Trial due to limited public access to detailed APT parameters from other trials, it serves as a pilot to demonstrate feasibility. As additional data on cost structure, success probabilities, and operational timelines from other APTs become available, we plan to extend the model to a larger sample of APTs across different disease areas and regions. Extending this framework to common, chronic, or infectious diseases would require strategic adaptations. Adjustments might include diversified funding sources, innovative royalty arrangements, or philanthropic participation tailored to diverse healthcare infrastructures and economic realities.

The conservative approach adopted to model competitor impacts, equating competitor entry with loss of exclusivity, may overestimate market share erosion. Real-world scenarios suggest new market entrants often coexist initially, particularly in diseases with limited effective treatments such as ALS, by serving distinct patient segments or clinical needs. Future analyses should explore less severe competitive scenarios to provide a more nuanced and realistic market outlook.

There is a risk that the fund will gravitate toward indications with the strongest investor appeal, potentially sidelining high-burden but less commercially attractive conditions. To address this, the royalty structure can be calibrated with tiered rates, carving our more generous returns for underfunded or priority indications. Additionally, each funding agreement can include clauses mandating affordable pricing or voluntary licensing in low- and middle-income countries, ensuring that breakthrough therapies remain accessible regardless of local economic constraints. Finally, future implementations could blend royalty funding with modest debt financing to lower the equity requirement, smooth cash flows, and extend the fund’s capacity to support a broader range of programs.

### Comparative analysis of alternative drug development financing models

In Table 4, we contrast our financial model with two non-traditional financing approaches (venture philanthropy and public–private partnerships) evaluating their advantages and disadvantages from both the drug developer’s and the investor’s perspectives.

**Table 4:** Comparison of proposed and alternative financing approaches.

(a) Drug Developer Perspective		
**Financing Approach**	**Advantages**	**Disadvantages**
**Adaptive Platform Trial Fund (Proposed)**	Efficient trial management Reduced upfront capital needs	Reduced flexibility in trial design Dependency on platform survival
**Venture Philanthropy**	Provides early-stage capital Validate the science and attract investors	Might require milestones and oversight Companies could face pricing issue
**Public–Private Partnerships**	Cost-sharing, risk reduction Smooth regulatory pathway	Multiple stakeholders; slower decision making Complex intellectual property and ownership
**(b) Investor Perspective**		
**Adaptive Platform Trial Fund (Proposed)**	Potentially high returns Risk diversification via multiple trials	Long investment horizon Inherently correlated assets by sharing platform
**Venture Philanthropy**	Can leverage additional capital Successful investment can be reinvested	Modest financial returns Substantial risk of failure
**Public–Private Partnerships**	Can mobilize more resources together than alone Solution for all stakeholder perspectives	Time-consuming legal/administrative process Public: Risk of paying twice if high drug price

The table summarizes key advantages and limitations of our proposed adaptive platform trial fund compared to alternative drug development funding methods, including venture philanthropy and public–private partnerships. This comparative analysis highlights the distinctive balance of efficient trial management and financial return provided by the proposed model.

Venture philanthropy is an investment approach in which disease-focused foundations provide capital to for-profit ventures under terms designed to achieve both patient-centered outcomes and modest financial returns—returns that are recycled into the foundation’s mission rather than distributed to outside investors [30]. For example, the Cystic Fibrosis Foundation’s (CFF’s) sale of its Kalydeco royalty rights for $3.3 billion not only validated this model but also demonstrated its transformative impact: almost every cystic fibrosis therapy on the market today traces its lineage to early CFF funding, underscoring how venture philanthropy can drive breakthroughs in diseases that are otherwise commercially neglected. Nonetheless, from a developer’s standpoint, these partnerships often hinge on meeting rigorous clinical and financial milestones, creating added operational burdens and potential delays if targets are not achieved [31]. Moreover, some foundations negotiate clauses that constrain pricing or patient-access terms once a drug is approved, which can limit a company’s revenue upside even when a program succeeds. From an investor’s standpoint, venture-philanthropic funds carry the same high-risk profile as traditional venture capital (especially when focused on early-stage rare-disease programs) meaning many bets may not pay off.

A public–private partnership (PPP) is a formally managed consortium of at least one nonprofit or government body and one for-profit company, convened to tackle drug development challenges that exceed the capacity of any single organization [32]. From a drug developer’s standpoint, PPPs offer significant cost-sharing benefits and mitigate development risk by pooling expertise and resources across sectors [33]. Engagement with regulatory agencies, notably the FDA’s advisory role in consortia, can clarify requirements early and streamline the approval pathway. However, these collaborations require consensus among diverse stakeholders, which can slow decision-making, and negotiating intellectual property rights and ownership can be intricate and time-intensive [34]. For investors, PPPs allow mobilization of far greater total resources than any single party could commit alone [33] and create structured forums where government, industry, academia, and patient advocates align on shared objectives [34]. Public investors can face a conflict of interest when therapies developed with taxpayer dollars are later sold at high prices, effectively causing the public to “pay twice” for the same treatment [35].

In contrast to these models, the Adaptive Platform Trial Fund uniquely combines the operational efficiencies of APTs with a royalty-backed financial structure. For developers, this means access to shared infrastructure, central data analytics, and reduced upfront capital requirements. For investors, it delivers a diversified, portfolio-based exposure to multiple drug candidates and the potential for high, royalty-linked returns. Although the model involves a long investment horizon and inherent asset correlation through a shared platform, its structured governance and strong profit potential align the interests of investors, drug developers, and patients.

## Conclusion

We examined the viability of a royalty-based financial structure designed specifically for APTs, based on the specific case of the HEALEY ALS Platform Trial that is being conducted by the Healey & AMG Center for ALS at Massachusetts General Hospital [13,14]. The platform clinical trial has properties that make it amenable to more efficient financial funding structures, including a regularity of schedule and a single disease target. This financial structure would fund 50% of the cost of each individual drug’s participation in the trial in return for a 7% royalty payment to investors.

We found that in the median case of our Monte Carlo simulations, there were 2 drug approvals (out of a possible 20), the NPV was $302.0MM (from an initial investment of $250MM), yielding an IRR of 28.4%, but with a 22.1% probability of total loss. Investments with such distributions may be most appropriate for sophisticated investors such as hedge funds, sovereign wealth funds, impact investors, and patient advocates engaging in venture philanthropy. In addition, by capping the fund’s commitment at 50%, we ensure substantial financial “skin in the game” for developers, aligning their incentives with accelerated timelines and disciplined cost control while preserving upside for investors.

However, to broaden appeal and minimize bias toward other indications, the model can incorporate tiered royalty rates, affordable-pricing/voluntary-licensing clauses for low- and middle-income countries, and modest debt overlays to diversify risk and extend support to underfunded disease areas. Although this pilot uses ALS-specific data due to limited public APT information, we intend to extend the framework to a larger sample of APTs across diverse diseases and regions as more data become available.

## Supporting information

S1 File Monte Carlo results. This file provides the full output of 10,000 Monte Carlo simulations, serving as the basis for the calculation of mean, standard deviation, median values, and the generation of all associated charts.
(XLSX)

S1 Fig Correlation map for the 20 regimens of the platform clinical trials. This Heatmap displays the pairwise correlations between regimens. Green cells represent platform-based systematic correlations of 20%, orange cells represent methodology-based high correlations of 80%, and white cells represent 100% self-correlation. This visual representation aligns with the HEALEY ALS Platform Trial data, where 20 out of 190 pairs identified as highly correlated.
(TIF)

S1 Appendix 1) Clinical trial costs, 2) sensitivity analysis. (DOCX)
