# Supplementary figures and images for: Financing drug development via adaptive platform trials
Source: PLoS One. 2025 Jul 2;20(7):e0325826. doi: 10.1371/journal.pone.0325826 (PMC12221166; doi:10.1371/journal.pone.0325826)

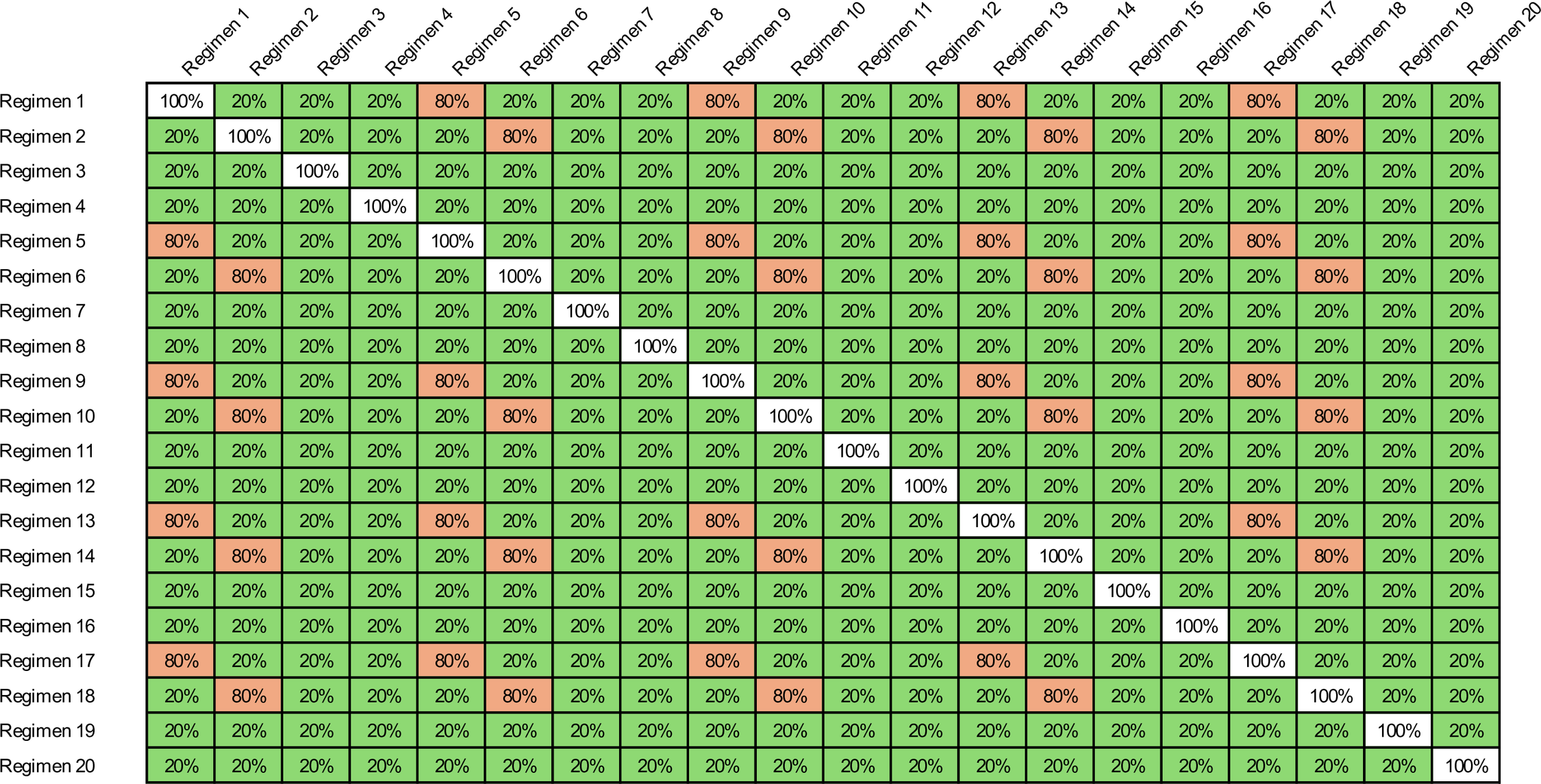

Supplement: S1 Fig — This Heatmap displays the pairwise correlations between regimens. Green cells represent platform-based systematic correlations of 20%, orange cells represent methodology-based high correlations of 80%, and white cells represent 100% self-correlation. This visual representation aligns with the HEALEY ALS Platform Trial data, where 20 out of 190 pairs identified as highly correlated. (TIF) [file pone.0325826.s002.tif]
