# Supplementary material for: Financing drug development via adaptive platform trials
Source: PLoS One. 2025 Jul 2;20(7):e0325826. doi: 10.1371/journal.pone.0325826 (PMC12221166; doi:10.1371/journal.pone.0325826)
Supplement: S1 Appendix — (DOCX) [file pone.0325826.s003.docx]

| **Table S1: Platform Build Cost (one-time cost), Design and Operations Cost ($MM)**  **by Number of Concurrent Regimens. (All translated to 2025 dollars)** | | | | | | | | | | |
| --- | --- | --- | --- | --- | --- | --- | --- | --- | --- | --- |
| **Platform Build Cost ($MM)** | | | | | | | | | | |
| **One-time Start Up Cost = 2.26** | | | | | | | | | | |
| **Designs (3 mo) and Operations (34 mo) Cost ($MM)** | | | | | | | | | | |
| **No. Concurrent Regimens** | 1 | 2 | 3 | **4** | 5 | 6 | 7 | 8 | 9 | 10 |
| **Design Costs** | 0.42 | 0.42 | 0.42 | **0.42** | 0.42 | 0.42 | 0.42 | 0.42 | 0.42 | 0.42 |
| **Operations – Fixed across Regimens** | 6.92 | 3.46 | 2.31 | **1.73** | 1.38 | 1.15 | 0.99 | 0.87 | 0.77 | 0.69 |
| **Operations – Regimen Specific** | 19.06 | 19.06 | 19.06 | **19.06** | 19.06 | 19.06 | 19.06 | 19.06 | 19.06 | 19.06 |
| **Total Costs Per Regimen** | 26.40 | 22.94 | 21.79 | **21.21** | 20.86 | 20.63 | 20.47 | 20.35 | 20.25 | 20.17 |
| In this paper, we assume 4 regimens will be grouped as one batch, so we assume a platform build cost of $2,256,004, a design cost of $424,979, and an operations costs of $1,731,144 (fixed across regimens) and $19,065,706 (regimen-specific). The cost is estimated in 2019 dollars and converted to 2025 dollars according to the BRDPI index. | | | | | | | | | | |

**S1 Table. Platform Build Cost (one-time cost), Design and Operations Cost ($MM) by Number of Concurrent Regimens. (All translated to 2025 dollars)**

**1. Clinical Trial Costs.**

To estimate the costs associated with each trial, we use 2019 cost data directly from the HEALEY ALS Platform Trial. Cost estimates are based on the average cost of four platform trial regimens (presented in 2019 dollars). This assumes an orally-administered drug that is tested in 160 participants with an infrastructure of 54 clinical study sites, and includes a 6-month follow-up of each participant in the Randomized Control Trial (RCT) period, plus an overlapping Active Treatment Extension (ATE) period (Active Treatment Extension, also known as Open Label Extension, allows participants to receive the active drug for the regimen to which they were assigned after completing the 24-week randomized controlled trial). Then, we inflate this figure by 20.2% inflation to yield current-dollar estimates based on the 2019–2025 Biomedical Research and Development Price Index (BRDPI) projections [13] (National Institutes of Health projects the BRDPI to grow at the rate of 3.3 percent in FY 2024, 2.7 percent in FY 2025, and 2.6 percent between FY 2026 and FY 2029). The platform trial costs can be broken down into several components: platform build, trial design, and trial operation.

Platform build costs are a one-time expense incurred during the start-up phase to establish the platform infrastructure. Trial design costs are associated with the early stage of trial development and primarily composed of personnel expenses for designing the clinical trial. Trial operation costs include the expenses of running and managing the trial. Each drug being tested in the platform trial infrastructure is called a regimen. A portion of the platform trial operation cost is shared across all regimens, i.e., as the number of concurrently processed regimens increases, the cost per regimen decreases. The remaining part of operation costs is fixed for each regimen. A more detailed breakdown of the platform build cost, regimen design cost, and regimen operation cost (adjusted to 2025 dollars) are presented in Table S1. According to the Healey and AMG Center for ALS, $2.26M ($1.90M in 2019 dollars) is required to build the platform, and $21.2M ($17.7M in 2019 dollars) is required to add one regimen to the platform trial, assuming 4 concurrent regimens over 37 months. After 2025, we incorporate a 2.6% inflation rate for annual costs, in line with BRDPI index projections.

**2. Sensitivity Analysis.**

In this section, we measure the sensitivity of key performance metrics, specifically, the default ratio, the net present value (NPV in $MM), the internal rate of return (IRR), and the number of drug approvals, to various parameters, such as the platform-based systemic correlation between trials, the peak market share, the discount rate, and the drug price.

**S2 Table. Sensitivity to Platform-based Systematic Correlation (Corr).**

The highlighted row corresponds to the base case scenario with a 20% platform-based systematic correlation.

| **Corr** | **Default Ratio** | **Median NPV** | **Mean (Std) NPV** | **Median IRR** | **Median Approval** | **Mean(Std.) Approval** |
| --- | --- | --- | --- | --- | --- | --- |
| 0% | 8.9% | 360.55 | 356.63 (276.84) | 30.7% | 2 | 3.0 (2.2) |
| 10% | 16.2% | 321.72 | 325.97 (321.99) | 29.6% | 2 | 3.1 (2.8) |
| **20%** | **22.1%** | **302.03** | **300.70 (341.22)** | **28.4%** | **2** | **3.1 (3.1)** |
| 30% | 29.9% | 256.68 | 268.54 (360.76) | 26.8% | 2 | 3.0 (3.5) |
| 40% | 33.8% | 215.31 | 251.94 (376.71) | 24.3% | 2 | 3.1 (3.9) |

**S3 Table. Sensitivity to Peak Market Share (Peak MS).**

The highlighted row corresponds to the base case scenario with a 55% peak market share.

| **Peak MS** | **Default Ratio** | **Median NPV** | **Mean (Std) NPV** | **Median IRR** | **Median Approval** | **Mean(Std.) Approval** |
| --- | --- | --- | --- | --- | --- | --- |
| 35% | 22.6% | 153.63 | 191.23 (276.10) | 22.7% | 2 | 3.0 (3.0) |
| 45% | 23.7% | 260.91 | 254.95 (316.35) | 26.6% | 2 | 3.0 (3.1) |
| **55%** | **22.1%** | **302.03** | **300.70 (341.22)** | **28.4%** | **2** | **3.1 (3.1)** |
| 65% | 21.7% | 329.21 | 318.66 (352.15) | 28.7% | 2 | 3.0 (3.1) |
| 75% | 22.1% | 342.66 | 329.71 (358.30) | 29.3% | 2 | 3.0 (3.0) |

**S4 Table. Sensitivity to Discount Rate (DR).**

The highlighted row corresponds to the base case scenario with a 13.69% peak discount rate.

| **DR** | **Default Ratio** | **Median NPV** | **Mean (Std) NPV** | **Median IRR** | **Median Approval** | **Mean(Std.) Approval** |
| --- | --- | --- | --- | --- | --- | --- |
| 9.5% | 22.2% | 591.96 | 588.68 (584.64) | 27.8% | 2 | 3.0 (3.1) |
| 11.5% | 22.9% | 396.08 | 415.80 (447.55) | 27.4% | 2 | 2.9 (3.0) |
| **13.69%** | **22.1%** | **302.03** | **300.70 (341.22)** | **28.4%** | **2** | **3.1 (3.1)** |
| 15.5% | 23.7% | 200.11 | 215.13 (279.15) | 27.6% | 2 | 3.0 (3.1) |
| 17.5% | 22.9% | 156.17 | 153.83 (222.75) | 28.2% | 2 | 3.0 (3.2) |

**S5 Table. Sensitivity to Drug Price (DP).**

The highlighted row corresponds to the base case scenario with a $158k drug price.

| **DP ($k)** | **Default Ratio** | **Median NPV** | **Mean (Std) NPV** | **Median IRR** | **Median Approval** | **Mean(Std.) Approval** |
| --- | --- | --- | --- | --- | --- | --- |
| 50 | 23.1% | -14.98 | -12.63 (104.97) | 12.6% | 2 | 3.0 (3.1) |
| 100 | 23.4% | 128.63 | 123.57 (203.14) | 21.2% | 2 | 3.0 (3.1) |
| **168** | **22.1%** | **302.03** | **300.70 (341.22)** | **28.4%** | **2** | **3.1 (3.1)** |
| 200 | 22.7% | 385.41 | 383.20 (407.07) | 30.7% | 2 | 3.0 (3.2) |
| 250 | 22.2% | 485.69 | 502.23 (497.89) | 34.2% | 2 | 2.9 (3.0) |
